# Comparison of Bayesian models to estimate direct genomic values in multi-breed commercial beef cattle

**DOI:** 10.1186/s12711-015-0106-8

**Published:** 2015-04-01

**Authors:** Megan M Rolf, Dorian J Garrick, Tara Fountain, Holly R Ramey, Robert L Weaber, Jared E Decker, E John Pollak, Robert D Schnabel, Jeremy F Taylor

**Affiliations:** Department of Animal Sciences, Oklahoma State University, Stillwater, OK 74078 USA; Department of Animal Science, Iowa State University, Ames, IA 50011 USA; Department of Animal Science, Kansas State University, Manhattan, KS 66502 USA; Division of Animal Sciences, University of Missouri, Columbia, MO 65211 USA; USDA, ARS, US Meat Animal Research Center, PO Box 166, Clay Center, NE 68933 USA

## Abstract

**Background:**

While several studies have examined the accuracy of direct genomic breeding values (DGV) within and across purebred cattle populations, the accuracy of DGV in crossbred or multi-breed cattle populations has been less well examined. Interest in the use of genomic tools for both selection and management has increased within the hybrid seedstock and commercial cattle sectors and research is needed to determine their efficacy. We predicted DGV for six traits using training populations of various sizes and alternative Bayesian models for a population of 3240 crossbred animals. Our objective was to compare alternate models with different assumptions regarding the distributions of single nucleotide polymorphism (SNP) effects to determine the optimal model for enhancing feasibility of multi-breed DGV prediction for the commercial beef industry.

**Results:**

Realized accuracies ranged from 0.40 to 0.78. Randomly assigning 60 to 70% of animals to training (n ≈ 2000 records) yielded DGV accuracies with the smallest coefficients of variation. Mixture models (BayesB95, BayesCπ) and models that allow SNP effects to be sampled from distributions with unequal variances (BayesA, BayesB95) were advantageous for traits that appear or are known to be influenced by large-effect genes. For other traits, models differed little in prediction accuracy (~0.3 to 0.6%), suggesting that they are mainly controlled by small-effect loci.

**Conclusions:**

The proportion (60 to 70%) of data allocated to training that optimized DGV accuracy and minimized the coefficient of variation of accuracy was similar to large dairy populations. Larger effects were estimated for some SNPs using BayesA and BayesB95 models because they allow unequal SNP variances. This substantially increased DGV accuracy for Warner-Bratzler Shear Force, for which large-effect quantitative trait loci (QTL) are known, while no loss in accuracy was observed for traits that appear to follow the infinitesimal model. Large decreases in accuracy (up to 0.07) occurred when SNPs that presumably tag large-effect QTL were over-regressed towards the mean in BayesC0 analyses. The DGV accuracies achieved here indicate that genomic selection has predictive utility in the commercial beef industry and that using models that reflect the genomic architecture of the trait can have predictive advantages in multi-breed populations.

**Electronic supplementary material:**

The online version of this article (doi:10.1186/s12711-015-0106-8) contains supplementary material, which is available to authorized users.

## Background

National Cattle Evaluation (NCE) has been employed within the US beef industry for over four decades [1] and is based upon mixed linear model methodologies [2]. NCE has provided purebred breeders with a valuable tool to increase genetic gains in many economically important traits, but this tool has been largely unavailable outside the seedstock sector. Meuwissen *et al.* [3] proposed a methodology, genomic selection (GS), which began to revolutionize NCE by allowing producers to reduce the generation interval through the avoidance of progeny testing. However, this methodology also has potential applications within the commercial beef cattle sector where most animals are crossbred, ancestry is often comprised of many breeds, and pedigree is unknown. Selection could be practiced with DGV in the absence of a formal genetic evaluation, or animals could be managed (i.e., in the feedlot) by determining their genetic potential for carcass and feed efficiency traits. Given the potential of these applications for increasing producer and operator profitability and better managing traits related to the consumer’s acceptance of beef, more research is needed to evaluate the efficacy of these technologies in crossbred cattle.

Many DGV methodologies have been shown to be effective when analyses are constrained to a single breed of cattle [4,5], and procedures to generate DGV have been most thoroughly tested in purebred populations due to the ease of DNA and phenotype (often expected progeny differences, known as EPD) collection and simpler population structure. However, admixture is prevalent in US commercial beef cattle populations, and DGV prediction models that were developed using registered animals from a single breed are likely to be ineffective outside of the purebred sector [6]. With few exceptions, genetic evaluation is currently performed only within the US registered sector, but some breed associations now include crosses such as British-Continental hybrids (i.e., LimFlex, Balancer, SimAngus, etc.) in NCE. There is also an increasing interest in performing genetic evaluation in the commercial sector (such as in a commercial feedlot) due to the increased amounts of genetic variation and greater number of phenotypes available for analysis, especially for traits for which phenotypes are a limiting factor in purebred seedstock (i.e., carcass traits). Simulation studies have suggested that while it may be difficult to generate precise across-breed DGV prediction models, these types of models could be effective across many breeds if the phase relationships between markers and QTL are preserved [7,8].

Recent studies [4,9] using Bayesian models to estimate DGV have found that Bayesian models have a small advantage over genomic BLUP (GBLUP), which incorporates REML estimation of variance components [10-12]. Those studies indicate that the advantages of some Bayesian models are primarily due to their ability to more appropriately model the architecture of QTL effects within the genome, especially for traits that possess large-effect QTL [13]. Moreover, these models can include and sometimes estimate a parameter, π, which represents the proportion of genotyped SNPs which are not associated with trait variation. While estimation of this parameter is problematic when the infinitesimal model holds, or when sample size is small, the approach allows an examination of the genetic architecture, which is particularly useful for oligogenic traits.

Development of DGV prediction models in admixed and crossbred populations has theoretical advantages, including an increased number of possible samples as compared to single-breed purebred populations and the ability to access data on animals in commercial and non-pedigreed populations. In addition, admixed populations have been effectively used to train prediction models in simulation studies [7] and shown to exhibit only small to moderate decreases in accuracy compared to purebred populations [14] provided that the breeds present in the validation population were also present in the training population [7,11,15]. Because the prediction accuracy of DGV is influenced by the extent of familial relationships between animals in the training and implementation populations, which may be reduced in composite populations [6,13], it is important to examine whether these approaches can be used in large crossbred populations.

Despite the preponderance of crossbred animals within the commercial US beef population, few studies have examined the development of multi-breed DGV prediction models using field data on crossbred beef cattle. The studies published to date have primarily focused on feed efficiency evaluations [16,17], although there is interest in more widespread applications for genomic selection in multi-breed beef cattle populations. A study by Kachman *et al.* [15] pooled purebred animals into multi-breed groups to compare prediction in multi-breed versus single-breed populations for weaning and yearling weight. They found that accuracies were similar for both across- and within-breed DGV, as long as animals for the breed being predicted were present in the training population. These studies and the interest in genotyping technology within the industry indicate that to ensure the broadest possible impact, we must begin to understand how genomic technologies can be applied in commercial cattle populations, not just in multi-breed purebred populations, for which pedigree is unknown, and complex admixture is prevalent.

Another study by Weber *et al.* [18] compared genomic prediction in a multi-breed composite population with single-breed and multi-breed purebred predictions for growth and carcass traits that were generated using BayesCπ and found that training in multi-breed populations aided in the prediction of composite computations comprised of those breeds, but accuracies across all of the populations were fairly low. Although their results varied substantially across traits and breeds, breeds other than Angus and Hereford were sparsely represented, and some breeds were not present in both the training and validation populations, which may partially explain the lower overall predictive power across all 18 breeds and composite populations in the study.

We hypothesize that additional accuracy could be obtained in crossbred, multi-breed commercial beef cattle populations by using models that account for unequal SNP variances for traits with large-effect QTL. To evaluate this hypothesis, we generated genomic prediction models using various Bayesian approaches implemented in the GenSel software package to evaluate the amount of accuracy that may be lost in multi-breed prediction due to inadequate modeling of genetic architecture. This information could lead to a greater and more effective use of genomic tests for multi-breed DGV prediction in commercial cattle. In this paper, we discuss the potential of using genomic technologies in the commercial beef industry, either for selection (cow/calf producers) or for management (feedlot managers or stocker operators that sort cattle based on genetic potential), by developing and validating genomic prediction models for carcass traits in the absence of pedigree or purebred training data.

To achieve these objectives, we used a crossbred population of commercial steers and heifers from the National Cattleman’s Beef Association sponsored Carcass Merit Project (CMP) to evaluate the accuracy of DGV prediction models for carcass traits using various proportions of animals in training and validation populations and four different Bayesian prediction models.

## Methods

### Populations

A subset (n = 3360) of individuals that contributed to the CMP study, originally implemented to address issues of consumer dissatisfaction with their beef eating experiences, were chosen for genotyping based on the availability of observations for Warner-Bratzler Shear Force (WBSF), which is an objective measure of meat tenderness, and completeness of carcass records for all other traits. The selected sample represented five different sire breeds of taurine cattle (Angus n = 660, Charolais n = 702, Hereford n = 1192, Limousin n = 285, and Simmental n = 521). The design of the CMP and procedures for data collection were described by Minick *et al.* [19]. All of the animals enrolled in the CMP were sired by registered bulls nominated by their respective breed associations (Angus, Charolais, Hereford, Limousin, and Simmental) whereas dams were from commercial herds. Angus- and Hereford-sired CMP progeny were most similar to a purebred population due to the sires being mated to commercial dams with Angus and Hereford ancestry, however, the Continental-breed sired progeny were most similar to crossbred commercial cattle populations due to mating of Limousin, Simmental and Charolais bulls to commercial cows with a high percentage of Angus ancestry [19,20].

### Phenotypic Data

USDA personnel recorded marbling score (MARB), hot carcass weight (HCW), fat thickness at the 12th and 13th rib interface (FT), and ribeye muscle area (REA) between 24 and 48 hours *post-mortem*. Steaks were vacuum-packaged and aged for 14 days before the collection of WBSF records at Kansas State University. Muscle, DNA, and white blood cells (WBC) were obtained for each animal from Texas A&M University under material transfer agreements (MTA) with each of the sample owners (American Angus Association, American Hereford Association, American Simmental Association, American International Charolais Association, and the North American Limousin Foundation). WBC samples were obtained at weaning, whereas muscle samples were obtained at harvest as carcass data were recorded and steaks were collected for WBSF analysis. Paternity and identification matching of DNA profiles from WBC and muscle samples were performed as part of the CMP protocol for all samples for which DNA was received, but were not performed for all animals within the project. Animals with paternity or identification errors were removed and DNA was re-extracted from muscle samples at the University of Missouri to ensure that genotypes and phenotypes correctly matched to the same animal. Genomic DNA was extracted from 2940 muscle samples by proteinase K digestion followed by phenol:chloroform:isoamyl alcohol extraction and ethanol precipitation [21]. The remaining 420 samples were not re-extracted and DNA provided by Texas A&M University was used since these samples had successfully passed paternity and identification verification. The number of phenotypes available for analysis for each breed and trait is in Table 1.

**Table 1:** **Number of phenotypes, means, and standard deviations for each breed and analyzed trait**

**Trait** ^**1**^	**Angus**	**Charolais**	**Hereford**	**Limousin**	**Simmental**	**Total**
WBSF (kg)	651 3.7 ± 0.8	695 4.4 ± 0.8	1095 4.8 ± 1.1	283 4.3 ± 1.0	516 4.4 ± 1.0	3240 4.4 ± 1.0
REA (cm^2^)	644 82.1 ± 7.4	693 90.9 ± 8.7	1090 83.4 ± 9.1	276 102.3 ± 14.3	510 83.4 ± 10.1	3213 86.4 ± 11.1
MARB	644 564.2 ± 96.8	695 504.8 ± 65.3	1095 490.6 ± 72.3	276 458.1 ± 65.4	53 562.3 ± 77.1	2763 509.5 ± 83.9
FT (cm)	611 1.4 ± 0.4	693 1.1 ± 0.4	1057 1.5 ± 0.5	276 1.1 ± 0.6	509 0.9 ± 0.6	3146 1.3 ± 0.5
HCW (kg)	644 357.0 ± 32.0	695 360.8 ± 37.7	1095 365.8 ± 33.6	276 362.2 ± 33.7	509 344.4 ± 41.7	3219 359.3 ± 36.3
YG	627 3.1 ± 0.6	689 2.5 ± 0.6	1095 3.2 ± 0.8	249 2.0 ± 1.1	510 2.8 ± 0.8	3170 2.9 ± 0.8

^1^WBSF, Warner-Bratzler Shear Force; REA, Ribeye Muscle Area; MARB, Marbling score; FT, Backfat Thickness; HCW, Hot Carcass Weight; YG, Yield Grade.

### Genotypic data

All CMP samples were genotyped using the Illumina BovineSNP50 BeadArray [22], which assays 54 001 SNPs. A custom Illumina GoldenGate assay (additional details are in [20]) was used to generate genotypes for an additional 96 putative SNPs located within 186 kb of *calpastatin (CAST)* and *calpain-1* (*CAPN1)* genes. All genotypes were called using Illumina GenomeStudio software. SNP locations were obtained using UMD3.1 build coordinates [23] and data were filtered for quality control. Animals were removed from analysis if their overall call rate was less than 95%. Filtering removed SNPs with a call rate less than 0.89 (to include all commercialized SNPs for tenderness), and minor allele frequency (MAF) less than 0.01, leaving 40 645 SNPs for analysis on 3240 animals (Angus n = 651, Charolais n = 695, Hereford n = 1,095, Limousin n = 283, and Simmental n = 516). FastPHASE v1.2.3 [24] was used to phase genotypes and impute the 0.89% of missing genotypes.

### Models

Across-breed DGV prediction models were developed for traits recorded in the CMP using four Bayesian methodologies (BayesA, BayesB95, BayesC0, and BayesCπ) that are implemented in the GenSel software package [25] developed at Iowa State University and widely used for GS [4,5,9]. Each trait (WBSF; REA; MARB; FT; HCW, percent cooking loss, %CL, and yield grade, YG) was analyzed using 160 000 Markov chain Monte Carlo (MCMC) iterations (10 000 for burn-in) and each model was parameterized using starting values estimated as weighted means of the within-breed residual and additive genetic variances that were obtained by applying REML to the GBLUP analysis (as in the GBLUP for WBSF reported in [20]), except where otherwise noted. Because estimates of the distribution of QTL effects were available for each trait from the GBLUP analyses (SNP allele substitution effects were estimated by regression on DGV according to [6]), this knowledge was used to define starting values for π, the proportion of markers that do not influence each trait. Traits for which large-effect genes were detected were provided a BayesCπ starting value of 0.99 and those that more closely followed the infinitesimal model received starting values that ranged from 0.9 to 0.95.

For these models, SNP effects are assumed normally distributed conditional on the SNP variances. All SNP variances had scaled inverse χ^2^ priors [9]. When π is estimated from the data (BayesCπ), it has a uniform (0,1) prior distribution. For all other analyses (BayesA, BayesB95, and BayesC0), π was assumed to be known and was specified in the analyses. Parameter starting values for each analysis are in Table 2. BayesC in which π = 0 (BayesC0) was used because of its similarity to GBLUP. Like GBLUP, BayesC0 assumes that all SNP effects are drawn from a distribution with a constant variance and that all SNPs contribute towards the prediction of DGV, but unlike GBLUP it does not assume a known variance. BayesA (in which π = 0) was also used, and, like BayesC0, includes all markers in the prediction model. BayesA [3] differs from BayesC0 in that individual SNP variances are estimated for each locus. In fitting BayesB with an assumed π > 0 (in our case, 0.95), two Metropolis-Hastings (MH) iterations were used in each step of MCMC sampling to determine if a locus should be sampled in that iteration. Finally, we performed a BayesCπ analysis which assumes a constant SNP variance [9]. However, the posterior means of each SNP effect variance were shrunk inversely proportional to the frequency with which each SNP was included in the model over the MCMC chain which essentially resulted in SNP effect variances that were unique for each SNP. In the BayesA, BayesB and BayesC analyses, π was treated as known (0 for BayesA, 0.95 for BayesB95 and 0 for BayesC0), whereas in the BayesCπ analyses, π was estimated from the data.

**Table 2:** **Parameter starting values for BayesCπ, BayesC0, BayesB95, and BayesA analyses**

**Trait** ^**1**^	**Analysis**	**π**	**V**_**a**_	**V**_**e**_
WBSF (kg)	BayesCπ	0.99	0.416	0.624
	BayesC0	0	0.416	0.624
	BayesA	0	0.16	0.55
	BayesB95	0.95	0.16	0.55
REA (cm^2^)	BayesCπ	0.95	25.629	40.170
	BayesC0	0	25.629	40.170
	BayesA	0	21	44
	BayesB95	0.95	21	44
MARB (units)	BayesCπ	0.9	3500	2600
	BayesC0	0	3500	2600
	BayesA	0	3500	2600
	BayesB95	0.95	3500	2600
FT (cm)	BayesCπ	0.95	0.092	0.063
	BayesC0	0	0.092	0.063
	BayesA	0	0.011	0.136
	BayesB95	0.95	0.011	0.136
HCW (kg)	BayesCπ	0.9	373.06	571.19
	BayesC0	0	373.06	571.19
	BayesA	0	610	615
	BayesB95	0.95	610	615
YG (units)	BayesCπ	0.9	0.2049	0.2049
	BayesC0	0	0.2049	0.2049
	BayesA	0	0.035	0.358
	BayesB95	0.95	0.035	0.358

^1^WBSF, Warner-Bratzler Shear Force; REA, Ribeye Muscle Area; MARB, Marbling score; FT, Backfat Thickness; HCW, Hot Carcass Weight; YG, Yield Grade.

Because the data were pre-adjusted for mean and contemporary group effects estimated in a BayesCπ analysis, the model fit to the data was:

$$ {y}_i={\displaystyle \sum_{j=1}^k}{z}_{ij}{u}_j+{e}_i $$

where:

- *y*_*i*_ = phenotypes pre-adjusted for the mean and contemporary group effects for each trait,
- *k* = number of marker loci fit in the analysis,
- *z*_*ij*_ = allelic state (*AA* = 10, *AB* = 0, *BB* = −10) of animal i at marker j,
- *u*_*j*_ = random additive effect for marker j, and
- *e*_*i*_ = residual.

For these models, the posterior mean for each marker’s effect was influenced by its realized rate of inclusion in the model when prior assumptions are *u*_*j*_ ~ $\mathrm{N}\left(\ 0,{\sigma}_u^2\right)$ with probability 1 - π or *u*_*j*_ = 0 with probability π. The DGV for each animal in the validation set was obtained as the sum of all individual estimated marker breeding values (using the posterior means for all post burn-in samples) over all *k* markers estimated during training.

**Figure 1:**
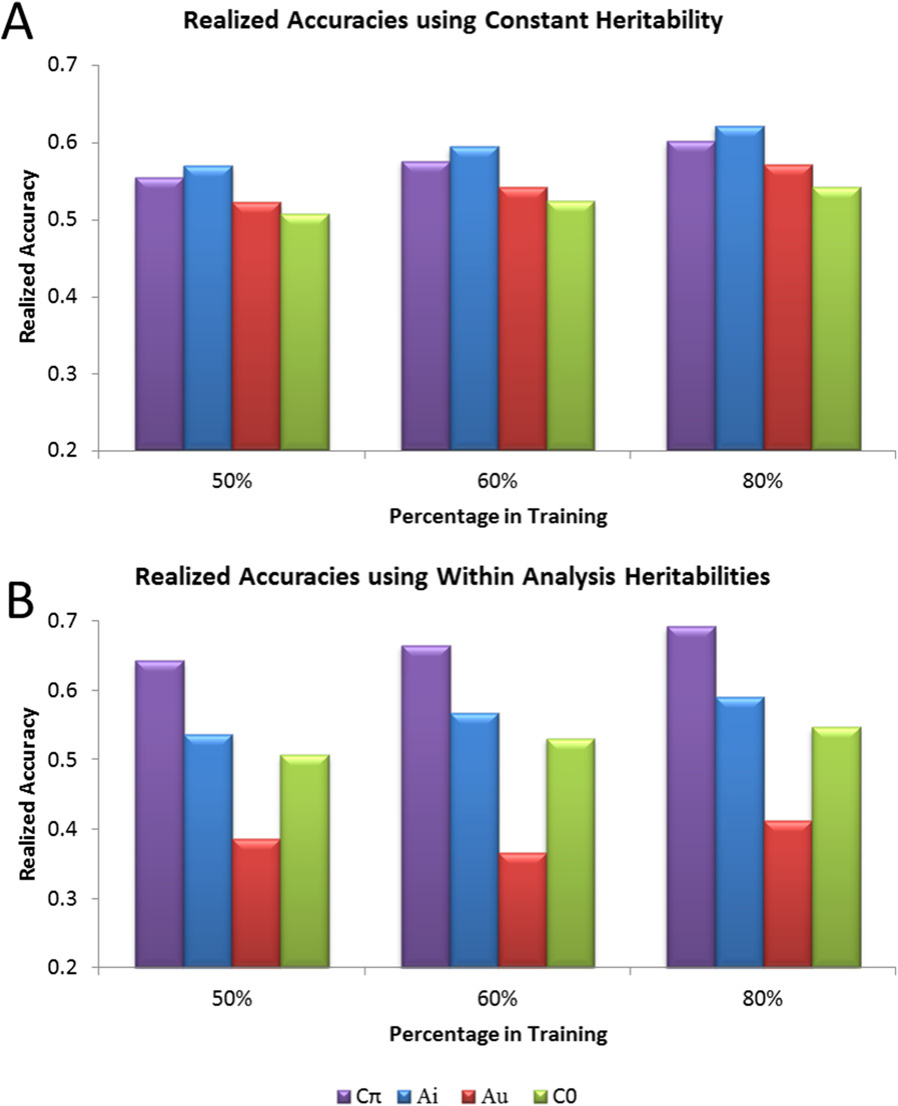
**Comparison of DGV accuracies achieved in validation from analyses with uninformed starting values (A**_**u**_ **) and informed starting values (A**_**i**_ **).** All analyses were completed for WBSF using a random sample of animals from the total sample for training and the remainder of animals used for validation. **Panel A** shows realized accuracies estimated using a heritability estimated from the BayesC0 best fit analysis. **Panel B** shows realized accuracies estimated using heritability estimates obtained within each respective analysis.

Starting values for additive genetic and residual variances for all analyses were generated as the weighted averages of each variance component across all within-breed GBLUP analyses for each trait for the BayesC0 and BayesCπ analyses. For all BayesA analyses, sensitivity of the estimated variance components to the starting values was noted (Figure 1) in the across-breed analyses when the weighted averages of variance components were used (A_u_), so the starting values were adjusted to reflect the posterior means from the BayesCπ analyses (A_i_), where the data overwhelmed the starting values. Sensitivity of BayesA and BayesB analyses to the starting values for variance components has previously been observed [9,26]. Identical training and validation data populations were analyzed by each model for all 20 bootstrap samples to ensure fair comparisons across analytical models. BayesB95 analyses were run exclusively on the best-fit data sets for which the allocation proportions into training and validation sets were determined to minimize the coefficient of variation for the correlation between DGV and phenotypes in the validation set. Contemporary groups were defined by the interaction between herd of origin, breed, sex, and harvest date and were modeled as a fixed effect in a single BayesCπ analysis including all animals for each breed and trait, and observations were then pre-corrected for these effects to generate the phenotypes used for subsequent analysis. This pre-correction step, rather than adjustment of records within each individual analysis, was necessary to allow the partitioning of animals into training and validation populations without the need to correct the data within each subpopulation for these effects.

GenSel reports correlations between phenotypes and DGV estimated in the validation population using the posterior means of SNP effects obtained in the training data analysis. The resulting correlation does not directly estimate the accuracy of the predicted DGV but is biased downwards because our input variables were phenotypes and not deregressed breeding values. In most cases, we report realized accuracies, calculated as $\frac{r_{\hat{g},y}}{\sqrt{h^2}}$ because $\sqrt{h^2}$ is the largest value that the correlation between phenotypes and breeding values can theoretically achieve. Within each breed, realized accuracies were estimated using the correlation from the GenSel analysis and heritabilities estimated from the corresponding within-breed GBLUP analysis using procedures outlined in McClure *et al.* [20]. Within-breed heritability estimates are in Table 3. Heritability estimates for cooking loss, kidney, pelvic, and heart fat, and internal fat percentages were not different from zero for all breeds and no further analyses were conducted for these traits.

**Table 3:** **Within-breed heritability estimates estimated by REML in GBLUP analyses**

**Trait** ^**1**^	**Angus**	**Charolais**	**Hereford**	**Limousin**	**Simmental**
WBSF	0.52	0.46	0.17	0.09	0.08
REA	0.61	0.21	0.23	0.55	0.29
MARB	0.51	0.57	0.49	0.41	0.87
FT	0.40	0.50	0.28	0.94	0.65
HCW	0.31	0.65	0.37	0.51	0.11
YG	0.39	0.50	0.23	0.79	0.45

^1^WBSF, Warner-Bratzler Shear Force; REA, Ribeye Muscle Area; MARB, Marbling score; FT, Backfat Thickness; HCW, Hot Carcass Weight; YG, Yield Grade.

### Allocation to training and validation populations

Animals were randomly assigned over 20 bootstrap replicates to training and validation populations regardless of breed. In cases where within-breed results were available [See Additional file 1: Table S1], they were generated by averaging the realized accuracies or correlations for a subset of animals of a single breed of sire within the validation population, rather than for the entire set of animals in the validation population. Breed of sire was not considered during allocation because the objective was to train and validate on multi-breed populations of animals comprised of commercially relevant US beef breeds. Animals were sampled using Matlab’s (Natick, MA) random number generator seeded by the CPU clock time to prevent identical assignments across replicates. If animals were partitioned into training and validation populations so as to minimize the extent of relatedness between individuals in these populations, realized accuracies of DGV would be expected to be minimized compared to those obtained with alternative methods of partitioning [4-6]. However, this approach was not feasible in this study because a large proportion of the data comprised single-progeny sire groups (>37%) and the total number of animals present as small half-sib families (≤10 progeny per sire) comprised 45% of the total data. Efforts to partition animals into training and validation populations based on genomic relationships resulted in trivial differences in DGV accuracies (data not shown). The structure of the data present in our analysis is similar to the type of data that would be expected in a commercial scenario. For example, if genotyping was routinely practiced at a feedlot where animals were tested upon arrival at the feedyard and allocated to pens based on their genetic propensity to achieve a desirable phenotype, no information (other than genotypes) would be available to account for relationships between animals. Consequently, our realized accuracies are likely to be less conservative than those found in studies that strove to reduce the extent of inter-family relationships between members of the training and validation sets.

A substantial number of different assignment proportions was tested (10% to 95% of the total dataset), to ensure a broad representation of sampling across the dataset. This extensive sampling procedure allowed us to ascertain at which point variance component estimates became most stable, and at which point the inclusion of additional animals into training yielded diminishing returns. In addition, because of the large sample size, the proportions tested in our analyses are representative of sample sizes in published cattle studies when we include sampling as little as 10% to 20% of the data (i.e., analyses using approximately 325 to 650 animals in training). No discernible increases in accuracy were realized after ~60 to 70% of the data were included in training. Therefore, we empirically identified the optimal proportion of data to assign to training (the best-fit analysis) from the analysis that produced the smallest coefficient of variation for the realized accuracies generated in the BayesCπ analyses of the 20 bootstrap replicates provided that > 50% of the data were used in training.

## Results and discussion

### Informed starting values

In a REML analysis, starting values for variance components are generally uninformed, but estimates will generally converge to a restricted maximum likelihood estimate regardless of the starting values. Upon convergence, they are then informed values. We employed a similar terminology to denote the starting values for our BayesA analysis (i.e., “uninformed” to represent starting values that were not based on any prior information generated by another Bayesian analysis, and “informed” for analyses which used starting values generated from a Bayesian analysis of the data that had reached convergence). In the BayesC analyses, the data overwhelmed the parameter starting values resulting in rapid convergence of the posterior means for variance components. The BayesA analyses were sensitive to starting values which required the use of starting values obtained from the BayesCπ analyses (Figure 1). Figure 1A illustrates the sensitivity of the BayesA analyses to the choice of starting values. A_u_ (red) represents the realized DGV accuracies achieved when variance component starting values were obtained from a weighted average across all breeds from a GBLUP analysis that incorporated REML variance component estimation [22] and A_i_ (blue) represents accuracies achieved when the means of the posterior distributions for additive and residual variance components from the BayesCπ analyses were used as starting values in a BayesA analysis. In Panel A, realized accuracies were normalized using a constant trait heritability that was estimated from the best fit BayesC0 analysis, and variation in realized accuracy reflects variation in the correlation between phenotype and DGV. Differences between using uninformed (A_u_) and informed (A_i_) variance component starting values were greater when the realized accuracies were estimated using heritability estimates produced within each analysis (Figure 1B). Finally, use of uninformed starting values for variance components led to the over-estimation of heritability, and systematic underestimation of realized accuracy.

### Heritability estimates and realized accuracy

Best-fit analyses were identified based on the set of 20 bootstraps for each model that produced the lowest coefficient of variation for the correlation between DGV and phenotypes in the validation set when > 50% of the data were used in training since using less data resulted in an increase in the sampling variance of the heritability and wide variation of the realized accuracy calculations [See Additional file 2: Figure S1A]. When a single heritability estimate based on a more robust sampling of data was used to estimate realized accuracies, results became more stable in the analyses using smaller subsets of the data [See Additional file 2: Figure S1B] and assessments of DGV accuracy could then be based solely on the estimated correlation between phenotype and predicted DGV within the validation set.

Correlations that were standardized using the heritability estimated from the BayesC0 analyses appeared to better reflect DGV accuracies (Table 4, last column). This was most notable for the BayesCπ analyses, which underestimated heritability compared to the other analyses in this study as well as REML estimates of heritability from GBLUP analyses [20]. Evidence of underestimation by BayesCπ can also be found in [See Additional file 2: Figure S1A]. When using the smaller heritability estimate from the BayesCπ analysis, the realized accuracies were sometimes outside the parameter space [0–1]. Correlations that were standardized using a heritability estimate obtained with all markers in the analysis (BayesC0) alleviated this issue [See Additional file 2: Figure S1B]. McClure *et al*. [20] reported a heritability of 0.25 across all breeds in this dataset. When compared to our heritability estimates (0.12 for BayesCπ, 0.26 for BayesC0, and 0.29 for BayesA), the analyses that fit all of the markers provided the closest estimates to the REML heritability estimate generated in the GBLUP analysis. This finding is likely due to the scaling parameters in the model as well as over-dependence on a few large-effect QTL, since this effect was only observed for traits for which very large SNP effects were detected. While this finding may vary from trait to trait, the heritability estimates obtained from the methodology that is most similar to REML/GBLUP (BayesC0) were stable over bootstrap samples, and appeared to be unaffected by GenSel’s scaling parameters in our study (unlike BayesCπ). Therefore, we recommend the use of the heritability estimate from the BayesC0 analysis to calculate the realized accuracies because it results in accuracy values that are similar in scale to those from a GBLUP analysis, which are the values presented in this manuscript, unless otherwise specified.

**Table 4:** **Parameters (π, h** ^**2**^ **)** ^**a**^ **, correlations between the DGV and phenotype** $\left({\boldsymbol{r}}_{\hat{\boldsymbol{g}},\boldsymbol{y}}\right)$ **from the best-fit analyses, and realized accuracies**

**Trait** ^**1**^	**n**_**t**_^**2**^	**n**_**v**_^**3**^	**Analysis**	**π**	**h** ^**2**^	${\boldsymbol{r}}_{\hat{\boldsymbol{g}},\boldsymbol{y}}$	**Realized accuracy**_**w**_^**5**^	**Realized accuracy**_**Cπ**_^**6**^	**Realized Accuracy**_**C0**_^**7**^
WBSF	2268	972	BayesCπ	0.9998	0.12	0.298	0.854	0.862	0.585
			BayesC0	0	0.26	0.276	0.547	0.796	0.541
			BayesA	0	0.29	0.314	0.586	0.906	0.616
			BayesB95	0.95	0.28	0.319	0.605	0.921	0.626
REA	1927	1286	BayesCπ	0.9931	0.32	0.336	0.600	0.594	0.585
			BayesC0	0	0.33	0.345	0.599	0.609	0.600
			BayesA	0	0.38	0.343	0.560	0.606	0.597
			BayesB95	0.95	0.36	0.344	0.573	0.608	0.599
MARB	1657	1106	BayesCπ	0.7432	0.62	0.595	0.757	0.756	0.762
			BayesC0	0	0.62	0.595	0.759	0.756	0.762
			BayesA	0	0.72	0.590	0.697	0.750	0.756
			BayesB95	0.95	0.67	0.592	0.722	0.752	0.758
FT	1887	1259	BayesCπ	0.9999	0.06	0.206	0.815	0.842	0.397
			BayesC0	0	0.27	0.267	0.517	1.091	0.514
			BayesA	0	0.11	0.242	0.746	0.990	0.467
			BayesB95	0.95	0.11	0.249	0.741	1.017	0.480
HCW	1931	1288	BayesCπ	0.9539	0.49	0.536	0.763	0.763	0.766
			BayesC0	0	0.48	0.543	0.785	0.772	0.776
			BayesA	0	0.63	0.536	0.677	0.762	0.765
			BayesB95	0.95	0.59	0.532	0.693	0.756	0.760
YG	2219	951	BayesCπ	0.9998	0.08	0.217	0.757	0.753	0.412
			BayesC0	0	0.28	0.264	0.502	0.914	0.500
			BayesA	0	0.13	0.256	0.714	0.888	0.486
			BayesB95	0.95	0.136	0.260	0.705	0.901	0.493

^a^Estimated as the means of posterior distributions over all post burn-in iterations.

^1^WBSF, Warner-Bratzler Shear Force; REA, Ribeye Muscle Area; MARB, Marbling score; FT, Backfat Thickness; HCW, Hot Carcass Weight; YG, Yield Grade.

^2^Number of individuals in the training population.

^3^Number of individuals in the validation population.

^4^Correlations reported are for best-fit analyses.

^5^Mean of realized accuracies calculated using the mean heritability estimate across all bootstrap samples within analysis.

^6^Mean of realized accuracies estimated using a heritability estimate produced from the best-fit BayesCπ analysis.

^7^Mean of realized accuracies estimated using a heritability estimate produced from the best-fit BayesC0 analysis.

### Bayesian model comparisons

Bayesian approaches have been shown to yield higher DGV accuracies than those produced by linear models when traits are influenced by genes of large effect [13]. In order to determine whether statistically significant differences in predictive capability between models exist, we performed paired t-test analyses of correlations (between DGV and phenotypes) for all analytical models across the 20 bootstrap replicates for each best-fit model (Table 5). While there were significant differences between models (p < 0.05, bonferroni-corrected p < 0.0014), those with the largest differences in means were for WBSF (BayesCπ *vs*. all other models and BayesC0 *vs*. all other models), FT (BayesCπ *vs*. all other models and BayesC0 *vs*. BayesA), and YG (BayesCπ *vs*. all other models). In one case, a 7% difference in accuracy was observed between the models with the highest and lowest accuracy (Table 4, last column). Overall, models that allowed unequal variances for SNP effects performed statistically better than those that assumed a constant SNP variance, particularly for traits that appeared to have large-effect QTL. The BayesA and BayesB95 analyses achieved the highest realized accuracies with no apparent penalty for including all of the markers in the analysis, presumably because the modeling of individual SNP variances allowed the effects for small-effect loci to be appropriately shrunk.

**Table 5:** **Results for paired t-test analyses of differences between mean correlations for all analytical models** ^**a**^

**WBSF**	**C0**	**A**	**B95**	**HCW**	**C0**	**A**	**B95**
Cpi	0.0247	−0.0170	−0.0231	Cpi	0.00234	0.0131	0.0062
	4.77	−3.28	−5.49		6.94	11.72	5.87
	<0.0001	0.0039	<0.0001		<0.0001	<0.0001	<0.0001
C0		−0.0418	−0.0479	C0		0.0108	0.0040
		−16.06	−17.24			9.17	3.10
		<0.0001	<0.0001			<0.0001	0.0059
A			−0.0061	A			−0.0070
			−4.41				−10.03
			0.0003				<0.0001
**REA**	**C0**	**A**	**B95**	**FT**	**C0**	**A**	**B95**
Cpi	−0.0096	−0.0080	−0.0092	Cpi	−0.0638	−0.0448	−0.0630
	−4.54	−4.08	−6.45		−9.80	−7.37	−6.70
	0.0002	0.0006	<0.0001		<0.0001	<0.0001	<0.0001
C0		0.0017	0.0005	C0		0.0190	0.0009
		4.25	0.49			8.58	0.11
		0.0004	0.6305			<0.0001	0.9133
A			−0.0012	A			−0.0181
			−1.36				−2.32
			0.1890				0.0317
**MARB**	**C0**	**A**	**B95**	**YG**	**C0**	**A**	**B95**
Cpi	0.0006	0.0077	0.0047	Cpi	−0.0493	−0.0413	−0.0452
	3.90	9.28	3.56		−10.00	−9.48	−11.98
	0.0010	<0.0001	0.0021		<0.0001	<0.0001	<0.0001
C0		0.0071	0.0041	C0		0.0081	0.0042
		8.00	2.87			3.17	1.65
		<0.0001	0.0099			0.0050	0.1147
A			−0.0030	A			−0.0039
			−2.90				−3.65
			0.0092				0.0017

^a^Results are across 20 bootstrap replicates for the best-fit model. The top line represents the mean difference between validation correlations for each model, the center value is the t-statistic for the test of no difference in model accuracies, and the bottom number is the corresponding p-value for the test.

**Figure 2:**
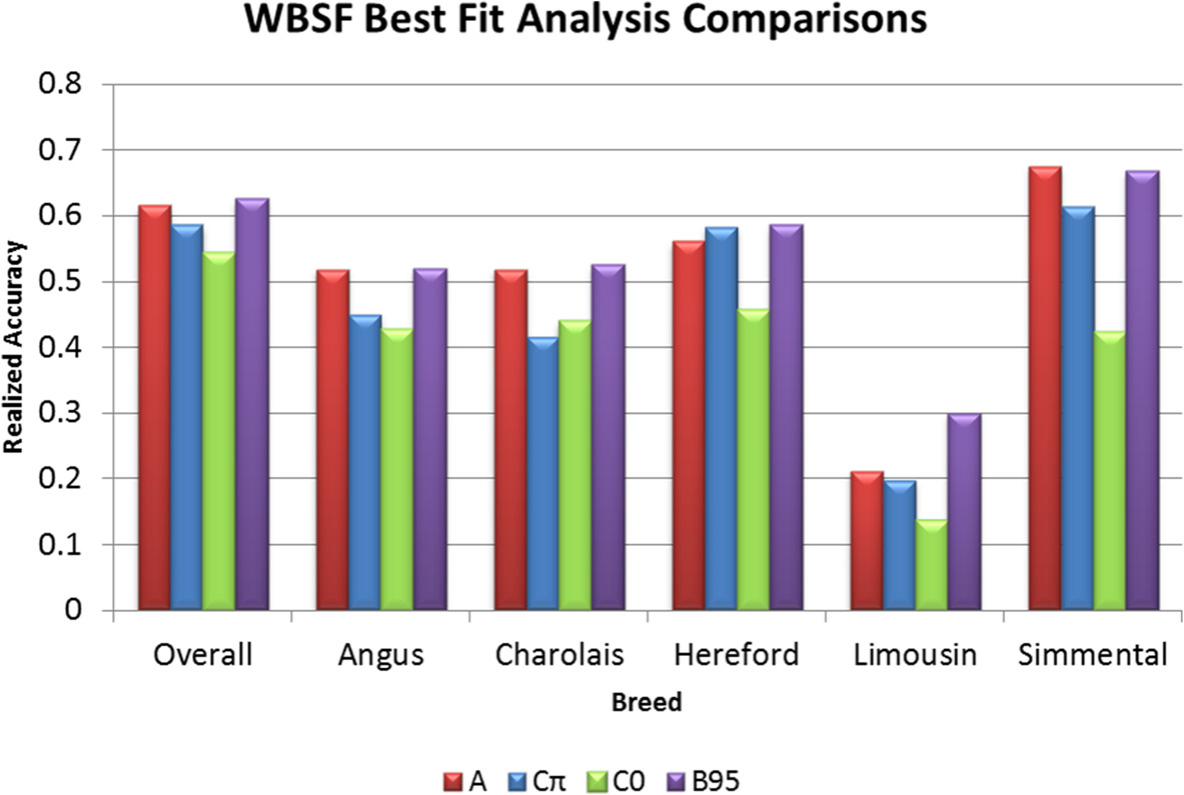
**Mean DGV realized accuracies for WBSF over 20 bootstraps for BayesA (red), BayesCπ (blue), BayesC0 (green) analyses, and BayesB95 (purple).** An across-breed estimate of heritability from the BayesC0 analysis was used for the calculation of overall accuracy and within-breed realized accuracies were calculated from within-breed estimates of heritability obtained through GBLUP.

**Figure 3:**
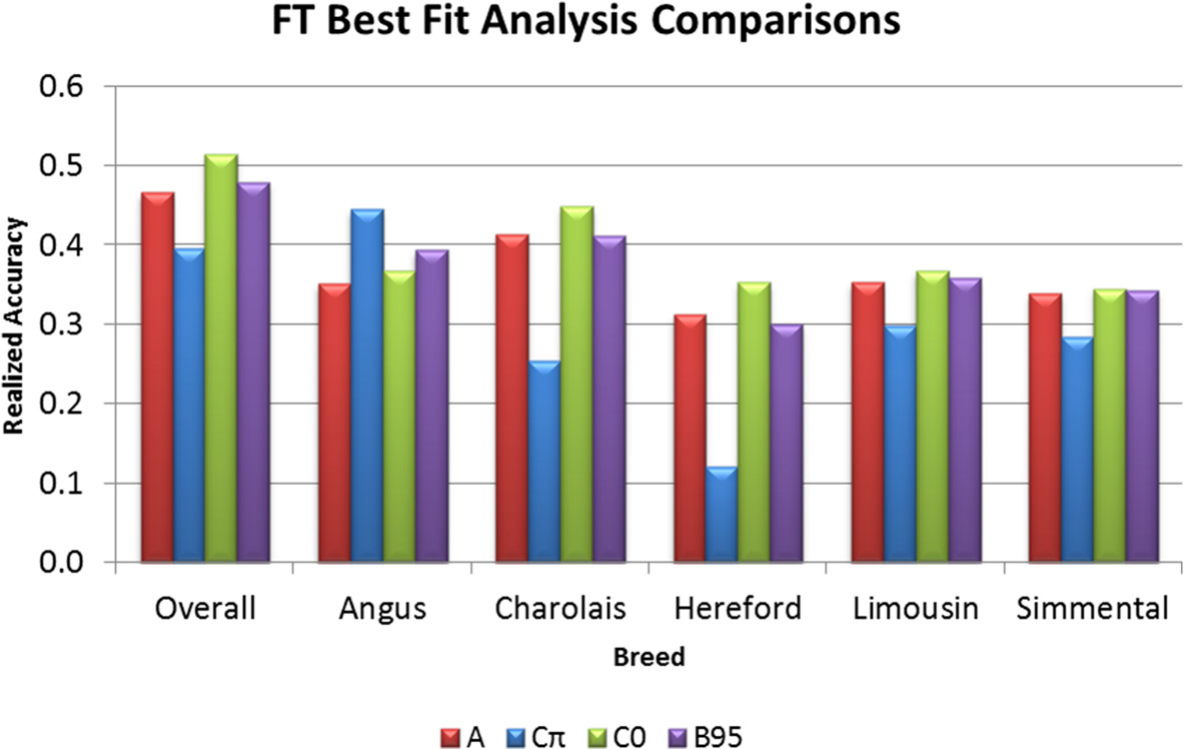
**Mean DGV realized accuracies for FT over 20 bootstraps for BayesA (red), BayesCπ (blue), BayesC0 (green) analyses, and BayesB95 (purple).** An across-breed estimate of heritability from the BayesC0 analysis was used for the calculation of overall accuracy and within-breed realized accuracies were calculated from within-breed estimates of heritability obtained through GBLUP.

Results for WBSF (Figure 2) were consistent with findings in the literature that indicate an advantage of Bayesian models that allow for unequal SNP variances for traits with large-effect QTL [13]. However, our analyses of FT did not confirm this advantage (Figure 3), although evidence for several genes of large effect was found for this trait in our population. BayesCπ was inferior to BayesC0 at predicting DGV for FT, probably because BayesCπ performed poorly in Hereford (Figure 3) and to a lesser extent in Charolais, which together comprised over 55% of the total FT dataset (Table 1). Results for YG were similar [See Additional file 2: Figure S2], presumably because of the strong dependence of YG on FT in the US beef grading system. The Angus and Hereford calves could be considered purebred commercial cattle, while the Continental breeds were a cross between Continental and Angus breeds; therefore, the superiority of the BayesCπ DGV prediction accuracies in Angus and the significantly reduced DGV prediction accuracy in Hereford with all of the Continental-sired calves having intermediate values suggests that the large-effect gene discovered for this trait is Angus-specific. The intermediate DGV prediction accuracy in Continental-sired calves reflects the fact that at least 50% (for some breeds this percentage can be greater than 50% due to “grading up” of Continental breeds within their registries) of chromosomes in these populations may be of Angus origin, resulting in the segregation of the FT QTL in these crossbred progeny. If the linkage phase relationship between SNP and QTL alleles is not preserved among breeds in the analysis, SNP effects will underestimate the contribution of QTL to DGV [27]. Therefore, phase relationships between SNP and QTL alleles must be preserved for this accuracy advantage to be realized within a multi-breed population.

**Figure 4:**
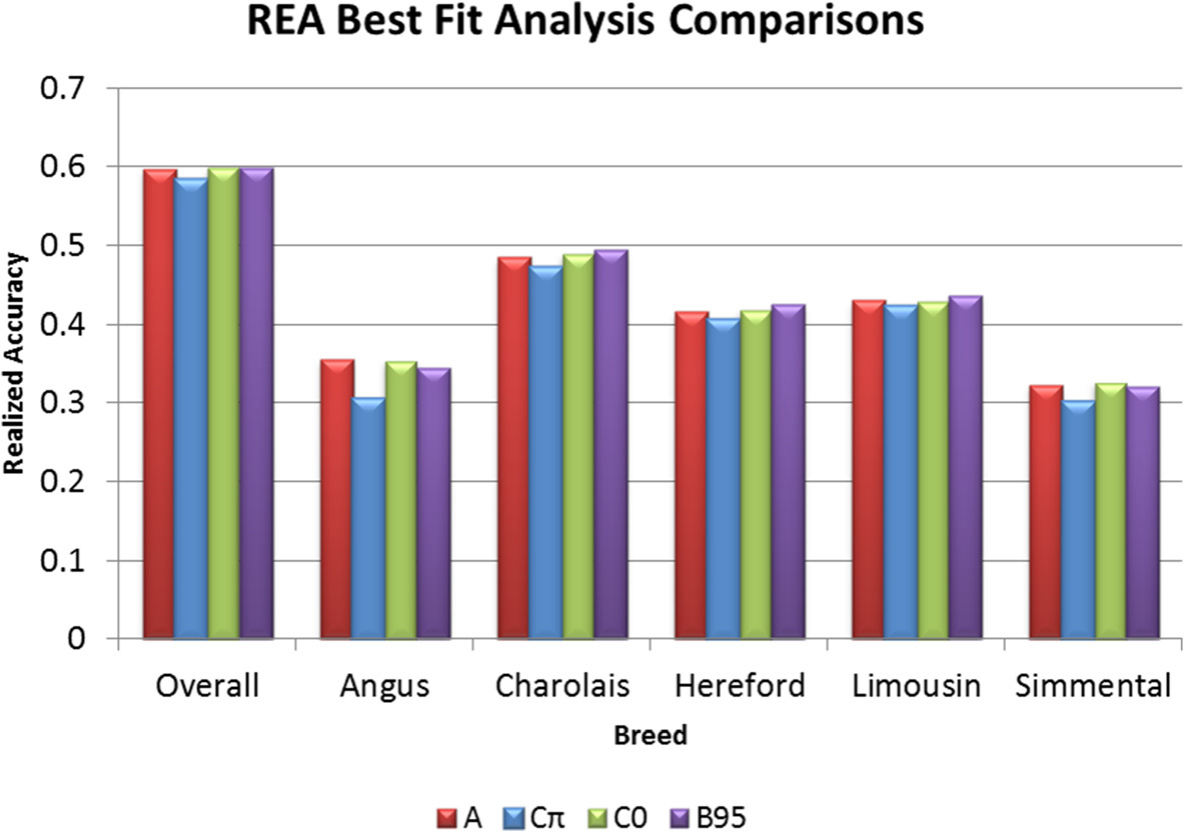
**Mean DGV realized accuracies for REA over 20 bootstraps for BayesA (red), BayesCπ (blue), BayesC0 (green) analyses, and BayesB95 (purple).** An across-breed estimate of heritability from the BayesC0 analysis was used for the calculation of overall accuracy and within-breed realized accuracies were calculated from within-breed estimates of heritability obtained through GBLUP.

It has been observed [28] that there are small differences between models that do not account for individual SNP variances (i.e., GBLUP and BayesC) and those that do (i.e., BayesA, BayesB) for traits that adhered to the infinitesimal model. In this study, for traits which did not appear to possess genes of large effect, such as REA (Figure 4), HCW [See Additional file 2: Figure S3] and MARB [See Additional file 2: Figure S4], DGV accuracies differed only slightly between analytical models (Table 5), regardless of the value of π, which indicates that constraining a proportion of loci to have no effect on a trait was far less important than the ability to assign individual locus SNP variances for the largest effect loci.

It has previously been observed that the predictive ability of a particular model depends on three attributes: effective population size, genetic architecture of the trait, and size of the training population [29]. Given these parameters, the fact that the BayesA and BayesB analyses (with π = 0 or 0.95, respectively) consistently performed well for all traits regardless of whether they were influenced by genes of large effect suggests that models which allow unequal SNP variances should be considered to be the “gold standard” for training GS models. We observed that these models were statistically better (Table 5) despite the statistical drawbacks that have been attributed to the current implementations of these methods [26]. Therefore, we qualify this statement by saying that this can only hold when well-estimated parameter starting values are available for the analysis.

### Random allocation

With no constraint on sample availability, the optimum number of animals to allocate to a training population will likely vary with the heritability of the trait and the effective population size, and may be anywhere from thousands to tens of thousands [30]. In this population of animals across all traits, random allocation of animals into training and validation populations proved computationally efficient and resulted in high accuracies of prediction (Table 4). Realized accuracies ranged from 0.41 (YG BayesCπ) to 0.78 (HCW BayesC0) and the highest accuracies were obtained for traits for which more markers were estimated to influence the trait. In a Holstein cattle dataset, Hayes *et al*. [31] observed that the accuracy of DGV predictions for traits with large QTL effects is higher than for traits with infinitesimal inheritance provided that the model used can account for the trait architecture. In this study, traits that involve genes of large effect tended to have the lowest realized accuracies. However, this may be influenced by the fact that the FT predictions (which also influence YG) were affected by a presumably breed-specific gene of large effect.

**Figure 5:**
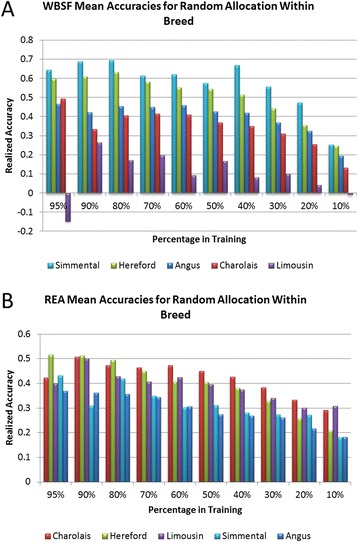
**Realized accuracies for DGV generated using BayesCπ and within-breed estimates of heritability for WBSF (Panel A) and REA (Panel B).**

Realized accuracies within each breed varied with the breed-specific heritability estimates for each trait. DGV accuracies estimated using breed-specific trait heritabilities are in Figure 5A for WBSF, Figure 5B for REA and in Additional file 2: Figures S5, S6, S7, and S8 for all other traits [See Additional file 2]. Presumably because of small sample size, accuracies that were estimated for Limousin tended to be the lowest (as shown for GBLUP in [6]), but this was not universally true (i.e., FT). A similar result was reported in [32], where animals that comprised the smallest proportion of the training population resulted in the lowest prediction accuracies. Breeds that were over-represented in the training set generally achieved the largest accuracies.

Best-fit analyses consistently used anywhere from 60 to 70% of the total data available for analysis. As the total data used in training increased, the realized accuracies in the validation population became increasingly volatile as the validation population sample size decreased [See Additional file 2: Figures S1, S9, S10, S11, S12, and S13]. Mean accuracy across the 20 bootstrap replicates was not different between analyses that used more than 60 to 70% of the data; however, the coefficient of variation increased substantially as the number of animals selected for validation became proportionally large or small. A similar phenomenon was observed by Erbe *et al*. [33] when anywhere from 73% to 95% of the total dataset was used for training. Consequently, using 60 to 70% of a dataset for training appears to be a viable heuristic for most moderately-sized datasets. It should be noted that for practical applications, all available records would be used in model training, and the assessment of prediction accuracy would be performed within the implementation population where performance is being predicted.

### Comparative analysis of realized accuracies

While studies in the literature that examined carcass trait DGV accuracies (with values between 0.07 and 0.31 [34] and −0.07 and 0.57 [35]) in very large multi-breed sheep populations (~5000 to 8000 animals) exist, only one other study to date has reported DGV accuracies for carcass traits in a large multi-breed beef cattle population [18], which ranged from 0.11 to 0.22. Direct comparison of accuracies between studies can be problematic, considering the diverse methods that are used to calculate DGV accuracy in the scientific literature. Our correlations (ranging from 0.206 to 0.595) exceeded those reported in [16] and our realized accuracies (ranging from 0.41 to 0.78 for all traits and analyses where realized accuracies were calculated using BayesC0 heritabilities) were greater than those reported in [17]; however, our study encompassed approximately five times the number of records as these studies and included carcass rather than feed efficiency traits. Our correlations were higher than those reported in [18] (0.11 to 0.22), possibly because our populations were more highly related than the crossbred multi-breed population and multi-breed purebred population in [18].

Most of the DGV accuracies that are reported for carcass traits in beef cattle come from studies on purebred populations. For example, Saatchi *et al*. [5] reported accuracies (expressed as the genetic correlation between the trait and its DGV) in Limousin (n ~ 2900, depending on the trait) and Simmental (n ~ 1700, depending on the trait) cattle of 0.56 to 0.59 for HCW, 0.98 for FT, 0.63 to 0.65 for MARB, 0.59 to 0.63 for REA, 0.53 for WBSF, and 0.62 to 0.67 for YG. Compared to our results, FT and YG accuracies were higher in their study, WBSF and REA were nearly equivalent, and MARB and HCW were higher in our study. However, it should be noted that pooling across breeds yielded more records as compared to [5] (approximately 1500 and 340 additional records as compared to the Simmental and Limousin analyses, respectively), but did not always result in superior accuracy given the multi-breed nature of the population. It should also be noted that our realized accuracies were less conservative due to random partitioning of animals into training and validation populations rather than to the minimization of the average relatedness among animals, since that was not feasible within the structure of our data. Saatchi *et al*. [4] also reported results for a similar analysis on Angus cattle, and the accuracies reported were 0.471 and 0.689 for HCW, 0.603 and 0.793 for FT, 0.690 and 0.817 for MARB, and 0.601 and 0.694 for REA, for K-means and random allocation, respectively. Comparing the results for random allocation to our results revealed that accuracies were greater in [4] than those achieved in our study (with the exception for HCW), which is probably due to the greater number of records (n = 3570), the use of deregressed EPD, and the single-breed population.

Goddard and Hayes [30] estimated that a minimum of approximately 50 000 animals would be necessary for reference populations in the case of lowly heritable traits (with a small effective population size of 100). It is unlikely that this number will be achieved in a single research study unless extensive pooling across breeds is performed or samples from industry are made available. This number of records has already been achieved in the Angus breed (http://www.angus.org/AGI/CelebrateHD50K.pdf), which demonstrates the potential that this technology holds as industry adoption increases. Commercial cattle populations will need a lower entry price point than seedstock operations, but the opportunity to achieve large numbers of animals for evaluation is much greater. However, it is likely that larger numbers of animals in the reference population will be necessary, since the effective population size will increase with the pooling of individuals across breeds which individually have effective population sizes of about 100 individuals.

Goddard and Hayes [30] also estimated that a reference population of at least 2000 to 3000 animals was needed to obtain prediction accuracies greater than 0.4 for moderately heritable traits (~0.3). When compared to [30], it would be reasonable to expect the maximum achieved accuracy to decrease slightly in a multi-breed application. However, we achieved accuracies of approximately 0.4 to 0.7 with moderately to highly heritable traits with a similar population size. It is well known that the extent of the relationship between training and implementation populations as well as the time since divergence of populations being predicted influences the accuracies obtained from genomic selection [6,13,32,36,37]. Presumably there is an underlying component of accuracy that is due to linkage disequilibrium, with the remainder being due to linkage [38]. In our study, the range of LD is expected to be attenuated due to the pooling of breeds [8]. In a commercial cattle population for which pedigree information is absent, such as in this study, it is likely that unknown pedigree relationships will bias the accuracies upward through the modeling of linkage information. However, it would be possible to take advantage of this increased accuracy due to linkage [38] as long as it is acceptable that the subsequent time-associated decay in accuracy from one generation to another is much greater than for a model that capitalizes on linkage disequilibrium alone [37]. We would generally expect prediction accuracies to increase as training population size increases, as has been noted for both theoretical [30] and real datasets [39].

### Industry impact and application

Potential industry impacts in the commercial sector have largely been ignored in the logical pursuit of enhancing predictive ability in purebred cattle, where GS has already been implemented. Nonetheless, tremendous potential exists to leverage those data and infrastructure investments to transfer this technology to the commercial beef industry. For this potential to be realized, foundational research must be completed to determine appropriate practices and applications.

The first, and most logical, place for the deployment of this technology in multi-breed populations is within the hybrid seedstock industry. Although these animals have pedigree data obtained from their respective breed organizations and possess EPDs from NCE along with their purebred counterparts, tremendous potential exists to refine prediction equations to maximize their efficacy in this industry sector. These predictions would likely primarily comprise two to three breed predictions (Angus or Red Angus and a Continental breed), and could set the stage for implementation in populations with higher amounts of admixture and unknown but highly variable breed composition.

It is also conceivable that commercial cattle could be tested upon entering a feedlot (if not earlier in life) and be sorted into groups by genetic potential to produce high-quality grade beef and/or for feed efficiency, or even by their susceptibility to common feedlot diseases, such as Bovine Respiratory Disease Complex. In this scenario, cattle could be fed and managed more appropriately to reduce waste and minimize labor costs associated with the monitoring and care of animals that are likely to become ill or moribund.

## Conclusions

By randomly allocating animals to training and validation populations, accuracies of DGV for the six traits studied here ranged from 0.40 to 0.78. The presence of large-effect QTL that do not segregate in all breeds is a significant limitation in multi-breed predictions. The best fit model depended on the genetic architecture of the trait and whether large-effect QTL were segregating. Models that were fit using BayesA consistently produced high DGV accuracies for all traits. In addition, models that can include unequal variances for individual SNPs produced higher accuracies than those that cannot for traits for which large-effect QTL are segregating. Using 60 to 70% of the total data for training provided the highest mean accuracy and the lowest coefficient of variation in accuracy across multiple bootstrap replicates.

Combined with the generally high prediction accuracies, these findings support the use of Bayesian models that allow the inclusion of unequal SNP variances for the implementation of genomic prediction in the US beef industry. This study provides the basis for further investigation of the use of genomic selection in the commercial beef industry, including the potential for its application in the cow/calf and feedlot sectors, provided that the cost/benefits ratio supports technology transfer to these sectors.

## Additional files

Additional file 1: Table S1. Contains correlations between the DGV and phenotypes for each trait and analysis. Additional file 2:
**Additional figures S1, S2, S3, S4, S5, S6, S7, S8, S9, S10, S11, S12, and S13 present data for each trait.**
