# Supplementary material for: Comparison of Bayesian models to estimate direct genomic values in multi-breed commercial beef cattle
Source: Genet Sel Evol. 2015 Apr 1;47(1):23. doi: 10.1186/s12711-015-0106-8 (PMC4433095; doi:10.1186/s12711-015-0106-8)
Supplement: Additional file 1: Table S1. — Contains correlations between the DGV and phenotypes for each trait and analysis. [file 12711_2015_106_MOESM1_ESM.docx]

**Table S.1: Mean correlations between the direct genomic value and phenotype (**$\boldsymbol{r}_{\hat{\boldsymbol{g}}\boldsymbol{,y}}$**) for animals in the validation population from the best-fit analyses for each trait within each breed of sire**^1^**.**

| Trait^1^ | Analysis | Overall | Angus | Charolais | Hereford | Limousin | Simmental |
| --- | --- | --- | --- | --- | --- | --- | --- |
| WBSF | BayesCπ | 0.2985 | 0.3232 | 0.2825 | 0.2401 | 0.0596 | 0.1706 |
|  | BayesC0 | 0.2758 | 0.3074 | 0.2976 | 0.1877 | 0.0414 | 0.1174 |
|  | BayesA | 0.3139 | 0.3727 | 0.3517 | 0.2312 | 0.0636 | 0.1876 |
|  | BayesB95 | 0.3194 | 0.3737 | 0.3566 | 0.2415 | 0.0905 | 0.1858 |
| REA | BayesCπ | 0.3360 | 0.2382 | 0.2188 | 0.1974 | 0.3158 | 0.1623 |
|  | BayesC0 | 0.3445 | 0.2756 | 0.2265 | 0.2025 | 0.3198 | 0.1754 |
|  | BayesA | 0.3431 | 0.2770 | 0.2242 | 0.2008 | 0.3202 | 0.1731 |
|  | BayesB95 | 0.3441 | 0.2681 | 0.2285 | 0.2054 | 0.3249 | 0.1720 |
| MARB | BayesCπ | 0.5953 | 0.4055 | 0.4033 | 0.4222 | 0.3893 | 0.4029 |
|  | BayesC0 | 0.5949 | 0.4051 | 0.4029 | 0.4212 | 0.3885 | 0.4001 |
|  | BayesA | 0.5903 | 0.4025 | 0.3908 | 0.4146 | 0.3751 | 0.3865 |
|  | BayesB95 | 0.5923 | 0.4047 | 0.3922 | 0.4176 | 0.3863 | 0.3777 |
| FT | BayesCπ | 0.1893 | 0.2533 | 0.1488 | 0.0376 | 0.2723 | 0.2482 |
|  | BayesC0 | 0.2500 | 0.2047 | 0.3081 | 0.1555 | 0.3084 | 0.2915 |
|  | BayesA | 0.2321 | 0.2248 | 0.2882 | 0.1350 | 0.2975 | 0.2921 |
|  | BayesB95 | 0.2492 | 0.2779 | 0.2902 | 0.1603 | 0.3485 | 0.2760 |
| HCW | BayesCπ | 0.5365 | 0.3013 | 0.3931 | 0.2900 | 0.3395 | 0.1048 |
|  | BayesC0 | 0.5348 | 0.3017 | 0.3865 | 0.2886 | 0.3299 | 0.0939 |
|  | BayesA | 0.5271 | 0.2817 | 0.3819 | 0.2833 | 0.3248 | 0.0847 |
|  | BayesB95 | 0.5320 | 0.2860 | 0.3927 | 0.2862 | 0.3362 | 0.1026 |
| YG | BayesCπ | 0.2171 | 0.2256 | 0.2296 | 0.0543 | 0.2279 | 0.1783 |
|  | BayesC0 | 0.2636 | 0.1951 | 0.3436 | 0.1510 | 0.2403 | 0.2196 |
|  | BayesA | 0.2560 | 0.1926 | 0.3405 | 0.1462 | 0.2562 | 0.2236 |
|  | BayesB95 | 0.2597 | 0.2202 | 0.3430 | 0.1368 | 0.2693 | 0.2229 |

^1^Within breed results were generated by averaging the realized accuracies for a subset of animals of a single breed of sire within the validation population, rather than for the entire set of animals in the validation population
