# Supplementary material for: Comparison of Bayesian models to estimate direct genomic values in multi-breed commercial beef cattle
Source: Genet Sel Evol. 2015 Apr 1;47(1):23. doi: 10.1186/s12711-015-0106-8 (PMC4433095; doi:10.1186/s12711-015-0106-8)
Supplement: Additional file 2: — Additional figures S1, S2, S3, S4, S5, S6, S7, S8, S9, S10, S11, S12, and S13 present data for each trait. [file 12711_2015_106_MOESM2_ESM.docx]

A

B

Figure S1: Comparison of realized accuracies estimated for WBSF BayesCπ analyses using heritability estimates produced in each analysis (Panel A) versus a constant heritability produced in the best-fit BayesC0 analysis.

Figure S2: Mean DGV realized accuracies for YG over 20 bootstraps for BayesA (red), BayesCπ (blue), BayesC0 (green) analyses, and BayesB95 (purple). An across-breed estimate of heritability from the BayesC0 analysis was used for the calculation of overall accuracy and realized accuracies within-breed were calculated from within-breed estimates of heritability obtained through GBLUP.


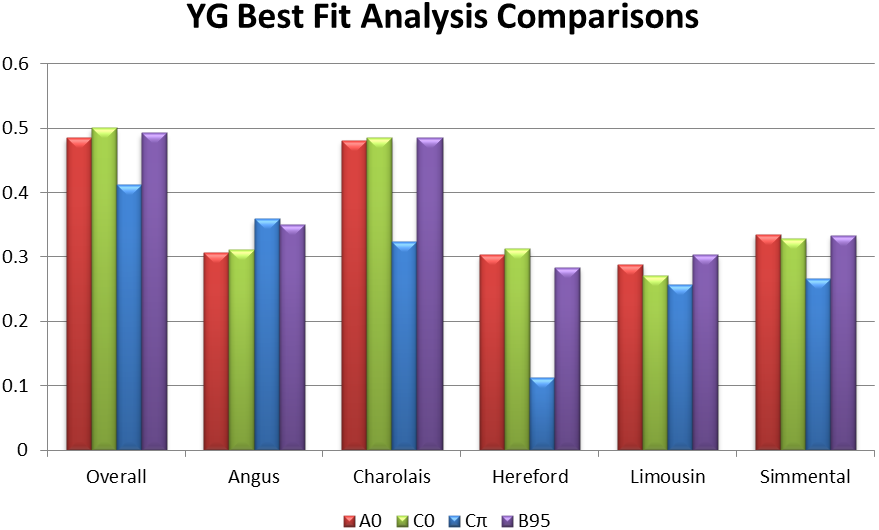


Figure S3: Mean DGV realized accuracies for HCW over 20 bootstraps for BayesA (red), BayesCπ (blue), BayesC0 (green) analyses, and BayesB95 (purple). An across-breed estimate of heritability from the BayesC0 analysis was used for the calculation of overall accuracy and realized accuracies within-breed were calculated from within-breed estimates of heritability obtained through GBLUP.


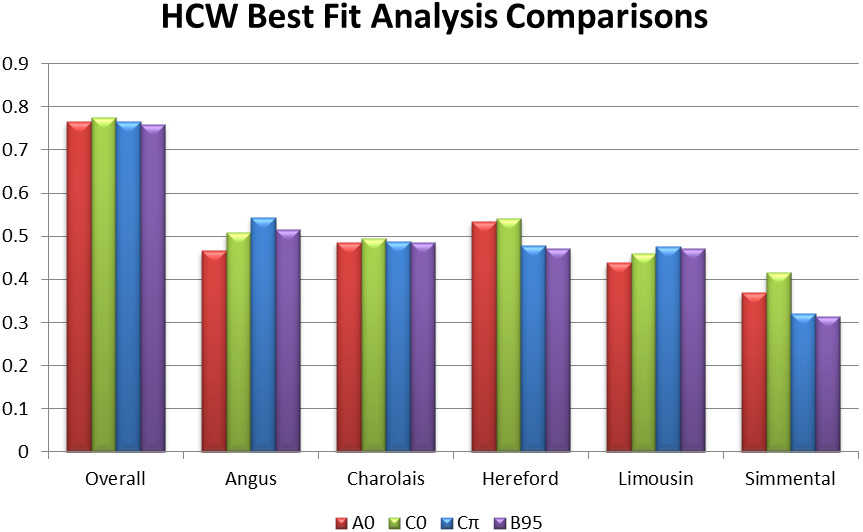


Figure S4: Mean DGV realized accuracies for MARB over 20 bootstraps for BayesA (red), BayesCπ (blue), BayesC0 (green) analyses, and BayesB95 (purple). An across-breed estimate of heritability from the BayesC0 analysis was used for the calculation of overall accuracy and realized accuracies within-breed were calculated from within-breed estimates of heritability obtained through GBLUP.


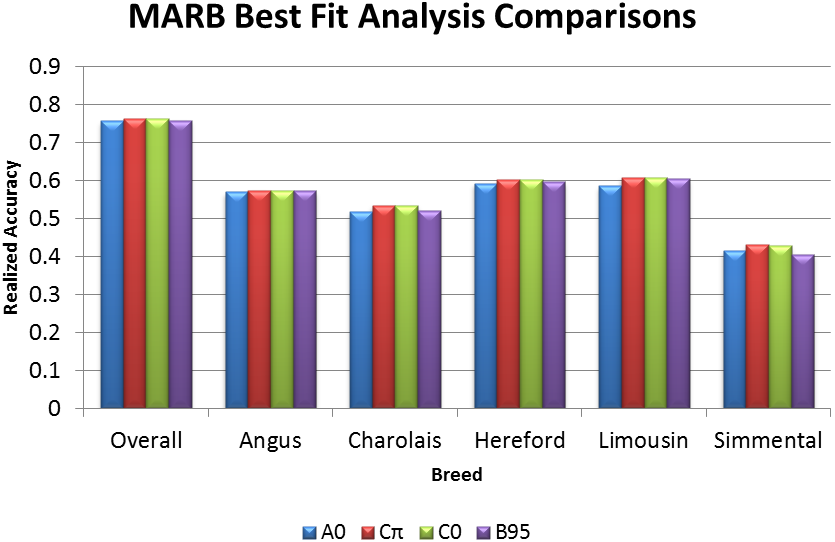

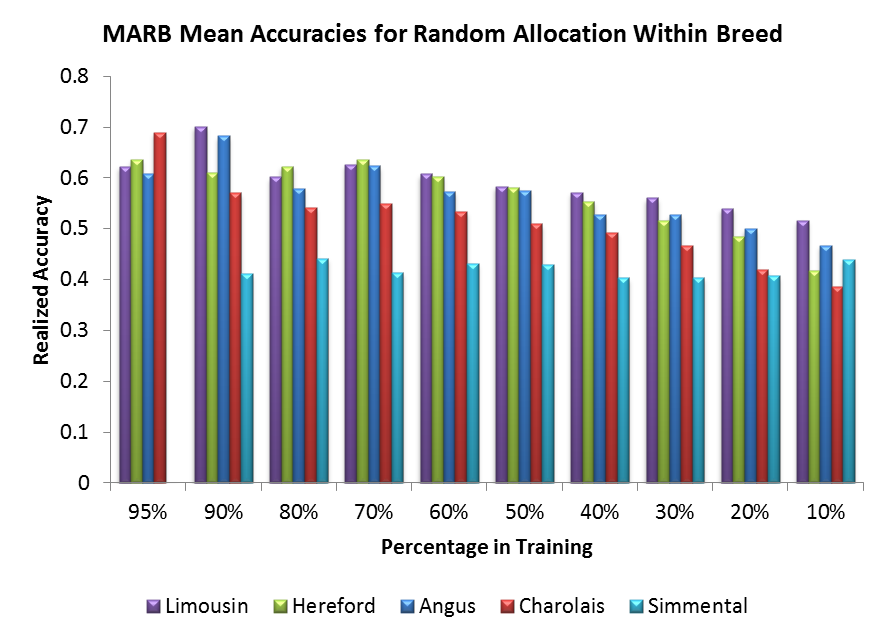


Figure S5: Within-breed realized accuracies for MARB using BayesCπ and random allocation.


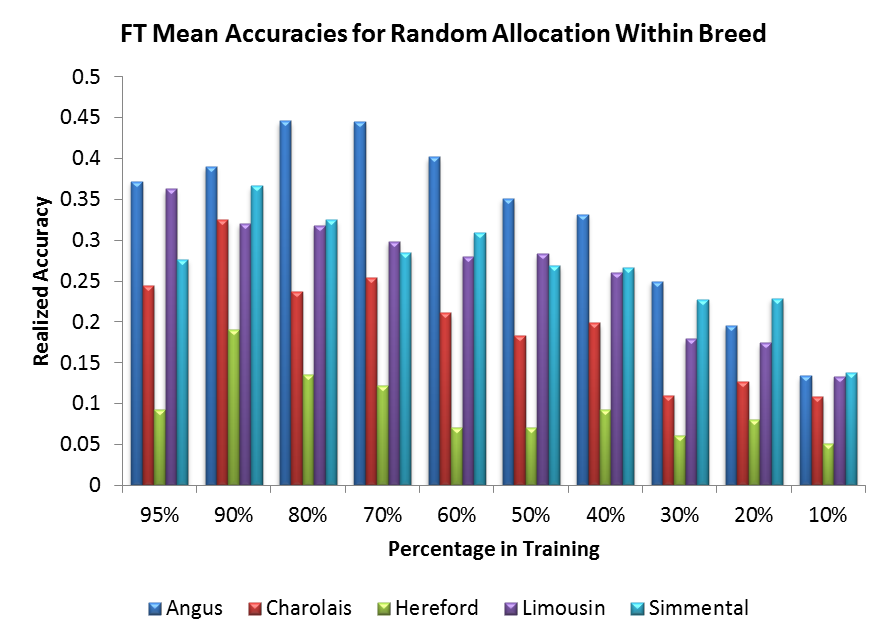


Figure S6: Within-breed realized accuracies for FT using BayesCπ and random allocation.


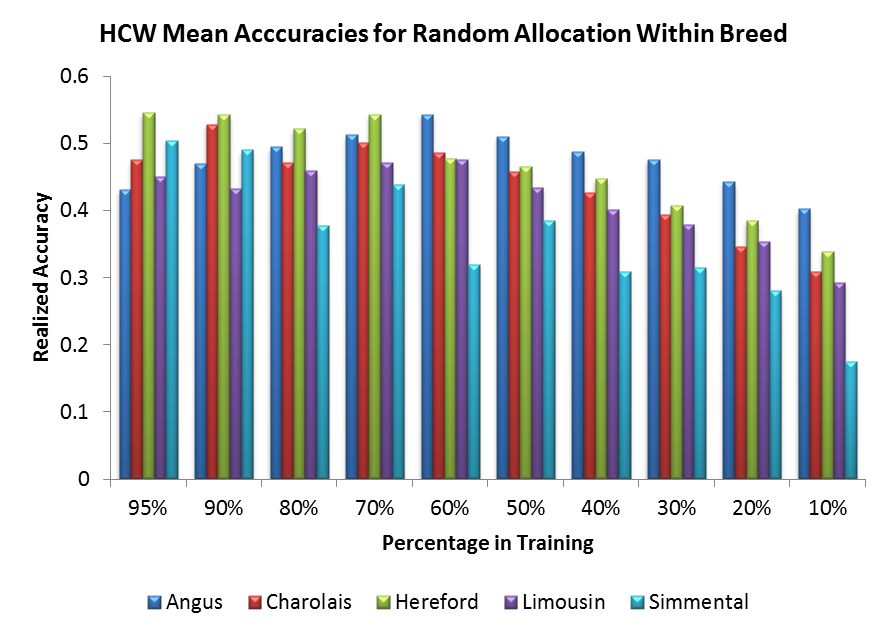


Figure S7: Within-breed realized accuracies for HCW using BayesCπ and random allocation.


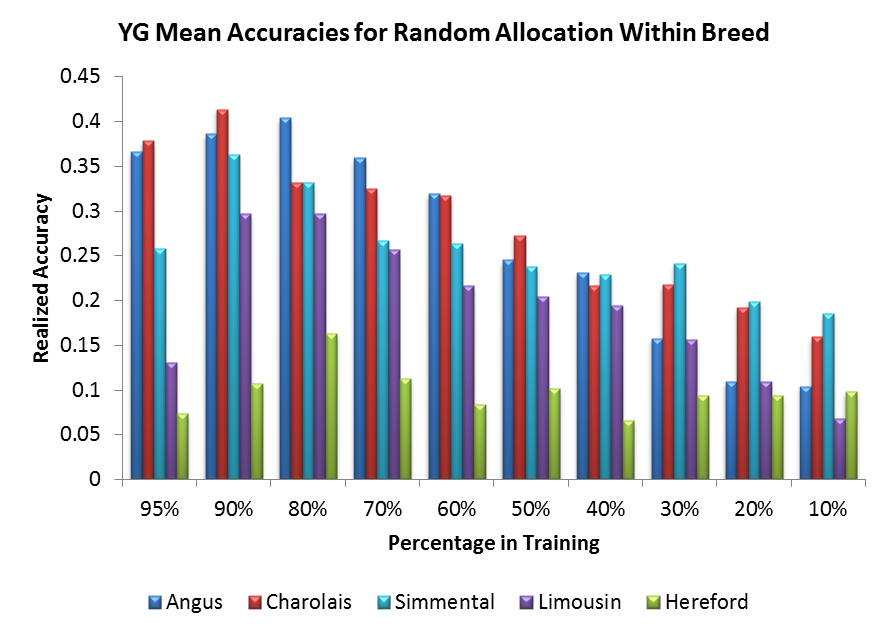


Figure S8: Within-breed realized accuracies for YG using BayesCπ and random allocation.


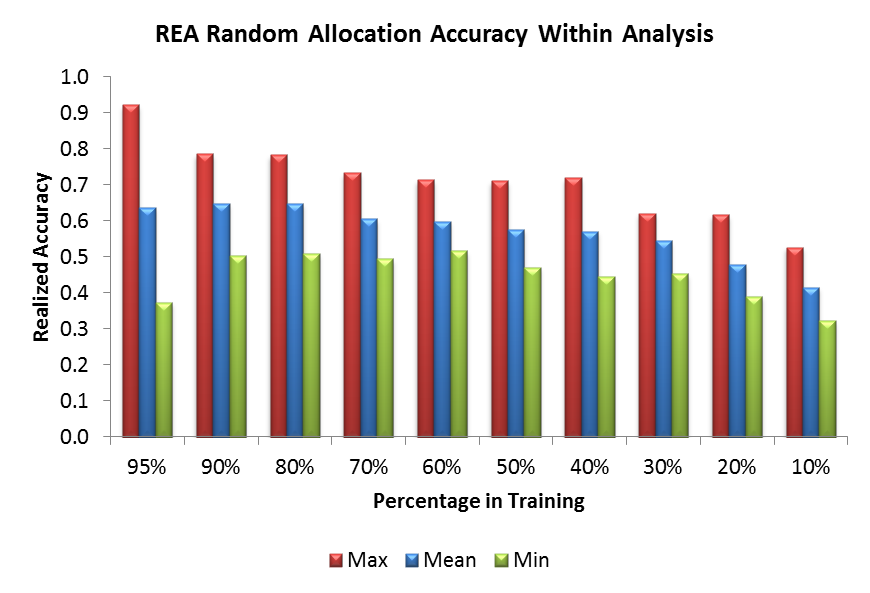

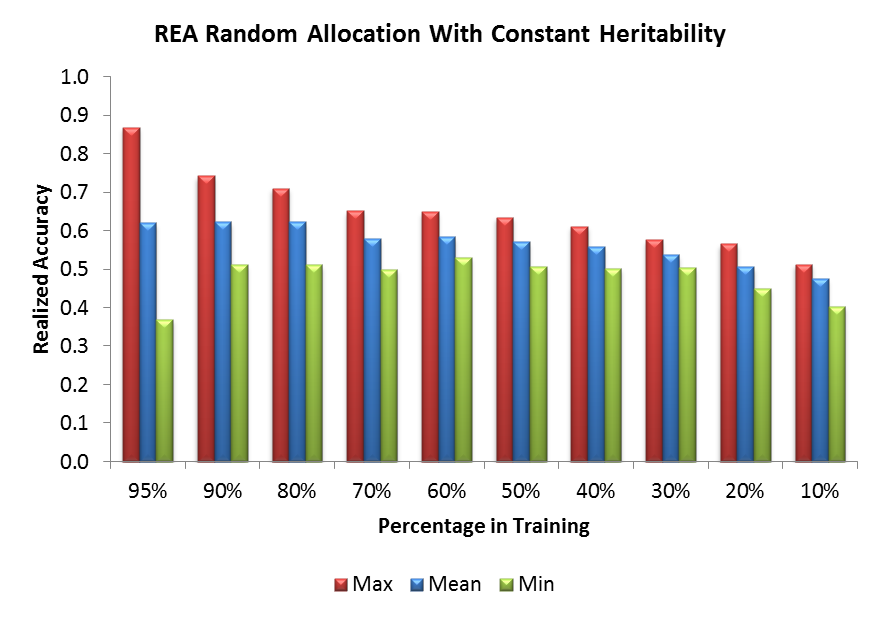


Figure S9: Comparison of realized accuracies calculated for REA BayesCπ analyses using within analysis heritability estimates (Panel A) versus a constant heritability defined as the mean of the best-fit BayesC0 analysis.

B

A


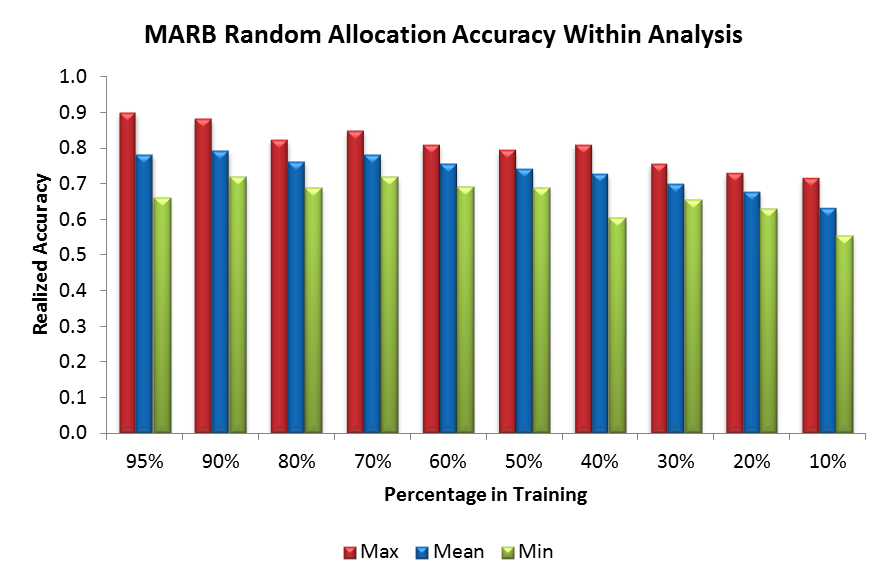

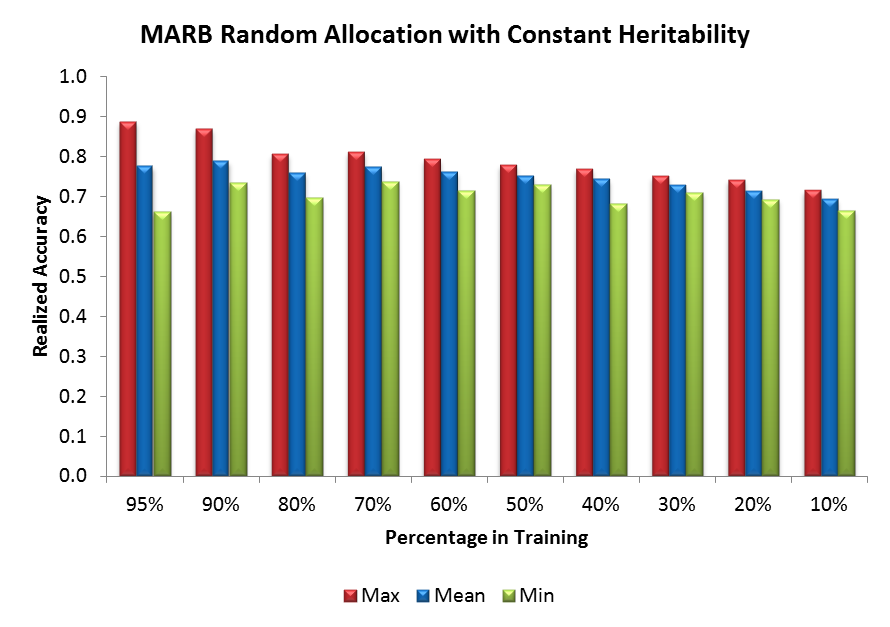


Figure S10: Comparison of realized accuracies calculated for MARB BayesCπ analyses using within analysis heritability estimates (Panel A) versus a constant heritability defined as the mean of the best-fit BayesC0 analysis.

B

A


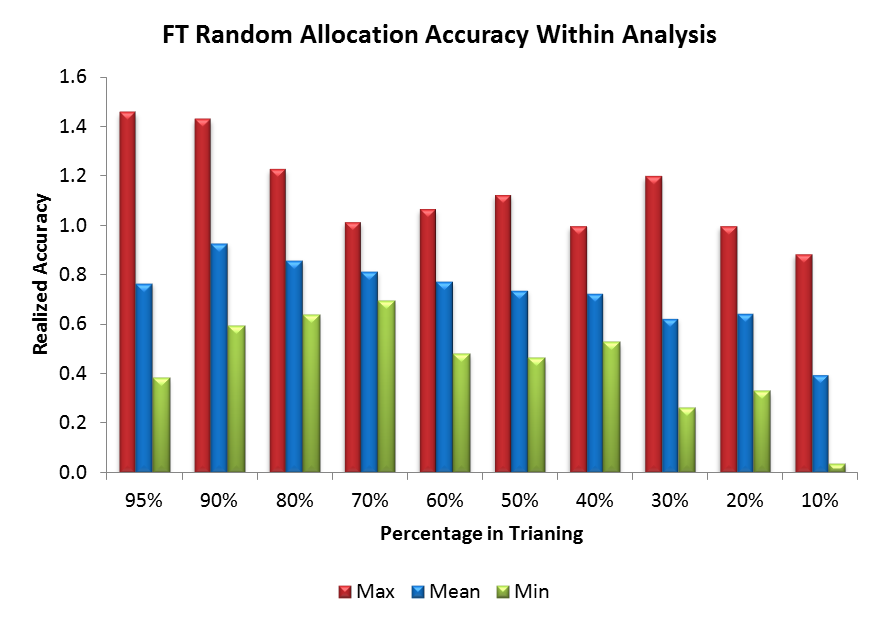

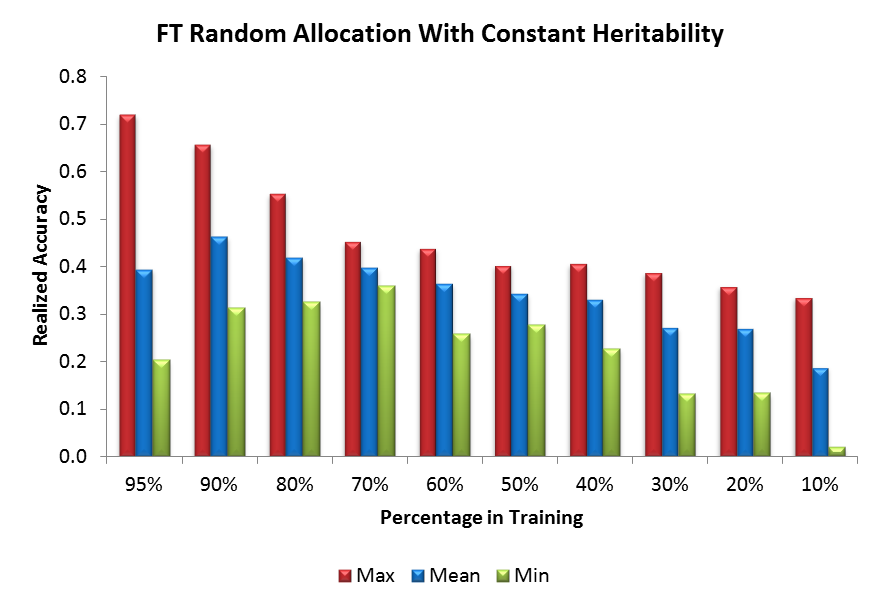


Figure S11: Comparison of realized accuracies calculated for FT BayesCπ analyses using within analysis heritability estimates (Panel A) versus a constant heritability defined as the mean of the best-fit BayesC0 analysis.

B

A


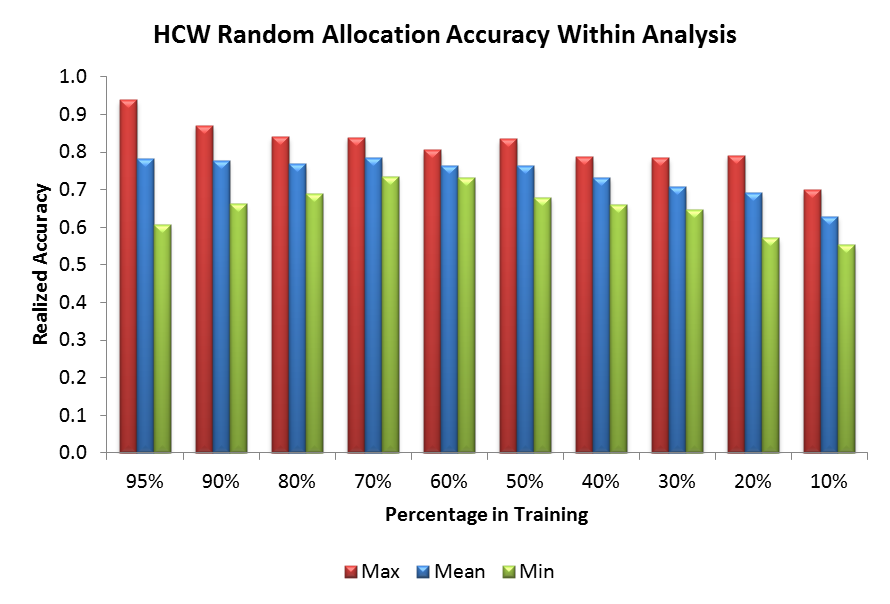

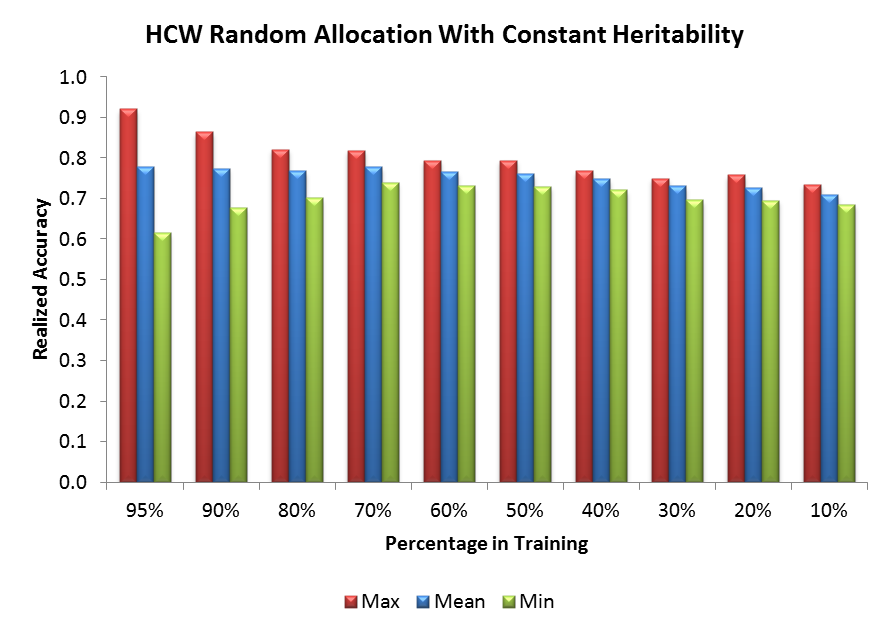


Figure S12: Comparison of realized accuracies calculated for HCW BayesCπ analyses using within analysis heritability estimates (Panel A) versus a constant heritability defined as the mean of the best-fit BayesC0 analysis.

B

A


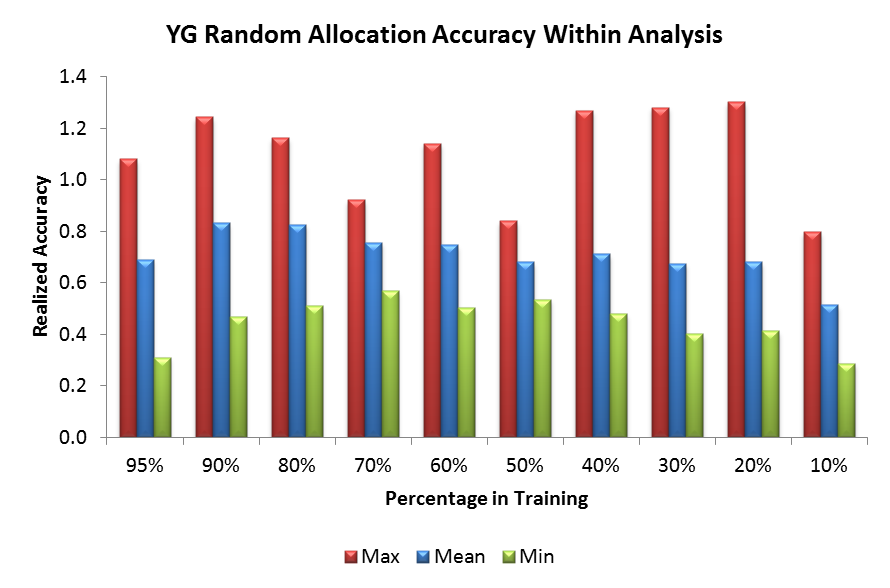

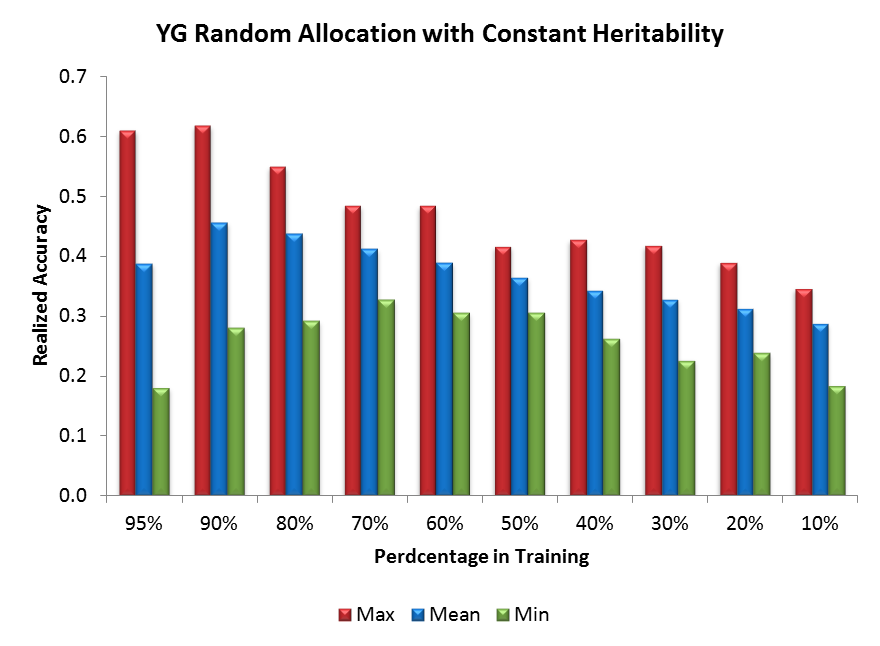


Figure S13: Comparison of realized accuracies calculated for YG BayesCπ analyses using within analysis heritability estimates (Panel A) versus a constant heritability defined as the mean of the best-fit BayesC0 analysis.

B

A
